# Sex Differences in Hemodialyzed Patients with End-Stage Kidney Disease—Data from a Large Polish Database

**DOI:** 10.3390/jcm15218490

**Published:** 2026-11-01

**Authors:** Wojciech Krasowski, Bartosz Symonides, Jacek Zawierucha, Wojciech Marcinkowski, Jacek Stanisław Malyszko, Jacek Lewandowski, Jolanta Małyszko

**Affiliations:** 1 Department of Nephrology, Dialysis and Internal Diseases, Medical University of Warsaw, 02-097 Warsaw, Poland; wojciech.krasowski@uckwum.pl; 2 Department of Internal Medicine, Hypertension and Vascular Diseases, Medical University of Warsaw, 02-097 Warsaw, Poland; bartosz.symonides@wum.edu.pl (B.S.); jacek.lewandowski@wum.edu.pl (J.L.); 3 Fresenius Medical Care, 60-118 Poznań, Poland; jacek.zawierucha@freseniusmedicalcare.com (J.Z.); wojciech.marcinkowski@fmc-ag.com (W.M.); 4 1st Department of Nephrology, Transplantology and Internal Medicine, Medical University Bialystok, 15-540 Bialystok, Poland; jacek.malyszko@umb.edu.pl

**Keywords:** sex, hemodialysis, end-stage kidney disease

## Abstract

**Background:** Chronic kidney disease is more prevalent in women, while more men than women start dialysis. Data on the sex differences in dialysis regarding comorbidities, dialysis adequacy, and access to transplantation are limited. Taking all these data into consideration, the aim of our study was to assess sex differences in an unselected large population of hemodialyzed patients in Poland. **Methods:** In a non-selected population of 5879 hemodialyzed patients (mean age 65.2 ± 14.2 years, 60% of males receiving hemodialysis), we assessed BMI (body mass index), comorbidities, status on transplantation list, pre-dialysis blood pressure and heart rate, hypertension presence, prevalence of apparent treatment resistant hypertension (aTRH), selected biochemical parameters, and data regarding HD (dialysis vintage, effective weekly dialysis time, eKT/V, ultrafiltration, interdialytic body weight gain unindexed and expressed as the percent of dry body weight in relation to sex). Propensity score matching was performed as an additional method to reduce baseline differences between men and women. **Results:** In the unselected population, men were younger, with lower BMI, lower prevalence of obesity, higher prevalence of hypertension and aTRH, had higher blood pressure with more hypotensive drugs, were more often dialyzed using arteriovenous fistula, lower dialysis vintage, higher time of dialysis, lower eKT/V, higher ultrafiltration and higher interdialytic body weight gain. After propensity score matching, age was not significantly different was well as prevalence of comorbidities (chronic heart failure, coronary artery disease, peripheral artery disease, cerebrovascular disease, diabetes). However, prevalence of hypertension and aTRH as well as blood pressure values were significantly higher in men over women. In addition, regarding parameters related to the dialysis, men more often had arteriovenous fistula as vascular access, lower dialysis vintage, higher time of dialysis, lower eKT/V and higher ultrafiltration and higher interdialytic body weight gain. **Conclusions:** Hemodialyzed women and men differed in several demographic, dialysis-related, and biochemical characteristics. Importantly, both sexes had the same access to active waitlisting list for kidney transplantation. These findings suggest that while biological and treatment-related differences between sexes persist in contemporary hemodialysis practice, access to transplantation appears equitable within this population.

## 1. Introduction

According to the Global Burden of Disease (GBD) 2023 study, chronic kidney disease (CKD) affects an estimated 93.1 million people in Europe, with more than half of cases occurring in Central and Eastern Europe and Türkiye [1]. CKD prevalence is higher in women than in men (11.8% vs. 10.4%), although biological factors unique to women, including pregnancy and hypertensive disorders of pregnancy, may contribute to this difference [2,3,4,5,6]. Despite the higher prevalence of CKD among women, men are more likely to receive kidney replacement therapy (KRT) [7,8,9,10]. Data on sex differences among patients receiving maintenance hemodialysis remain limited, particularly in Central and Eastern Europe. The DOPPS study identified important differences between women and men in demographic and clinical characteristics but did not include any countries from this region [7,8,9]. Registry-based analyses have primarily focused on sex differences in KRT incidence and prevalence, without detailed evaluation of comorbidities, dialysis-related characteristics, or attainment of treatment targets [2,10,11,12,13,14,15,16,17,18,19,20,21]. Although Weigert et al. reported sex differences in body weight, prescribed blood flow rate, single-pool Kt/V, hemoglobin concentration, and vascular access among hemodialysis patients from selected centers in Poland and Portugal, the study was limited by its relatively small sample size and restricted geographic coverage [11]. Furthermore, the third iteration of the International Society of Nephrology Global Kidney Health Atlas provided the most comprehensive overview of nephrology care in Central and Eastern Europe but did not report sex-specific data for this region [22]. In addition, in the recently published “Women’s Kidney Health in Focus: A Scientific Statement From the American Heart Association” in the section knowledge gaps and opportunities, improvement in the enrollment of women with adequate power to allow for sex-specific analysis and reporting of findings was advocated [23].

Therefore, given the limited evidence on sex differences in the hemodialysis population of Central and Eastern Europe, including Poland, we aimed to evaluate sex-related differences in a large, unselected national cohort of patients with end-stage kidney disease (ESKD) treated with hemodialysis, including access to kidney transplantation. We also assessed whether attainment of Kidney Disease: Improving Global Outcomes (KDIGO) and Kidney Disease Outcomes Quality Initiative (KDOQI) treatment targets differed between women and men [24,25,26]. In addition, using propensity score matching, we examined whether any observed sex differences persisted after adjustment for age, cardiovascular disease, and diabetes.

## 2. Patients and Methods

We conducted a retrospective analysis of a fully anonymized database comprising *n* = 5879 unselected, consecutive patients undergoing HD (mean age 65.2 ± 14.2 yrs., 60% of men) provided by the largest non-tertiary center for HD in Poland. The database was updated on 6 October 2022 and included averaged data from a complete 4-week period spanning from 29 August 2022 to 25 September 2022. The database included demographic data, the etiology of CKD, data on comorbidities, pre-dialysis BP (blood pressure) and HR (heart rate), data regarding HD. We analyzed the database updated on 6 October 2022, which included averaged data from the full 4-week period from 29 August 2022 to 25 September 2022.

### 2.1. Methods

We compared men vs. women in a non-selected group of patients and in sex groups matched according to age and concomitant diseases. The comparison included age, BMI (body mass index) dry body weight, bioimpedance (OH/ECW, where OH (overhydration) in L and ECW (extracellular water) in L were measured using a BCM (body composition monitor produced by Fresenius, Bad Homburg, Germany, measured before midweek dialysis every 6 weeks by protocol in FMC units), data on concomitant diseases (Charlson comorbidity index score, cardiovascular disease, diabetes mellitus), status on transplantation list, pre-dialysis BP and HR, hypertension presence, apparent treatment resistant hypertension (aTRH) presence, selected biochemical parameters as hemoglobin (Hb), ferritin, parathormone (PTH), and data regarding HD (dialysis vintage, effective weekly dialysis time, eKT/V, ultrafiltration (UF), ultrafiltration indexed over dry body weight (UFi), interdialytic body weight gain (IBWG) unindexed and expressed as the percent of dry body weight (IBWGi)). BP measurements were performed according to the current guidelines using validated sphygmomanometers. Biochemical parameters were assessed according to standard procedures.

We also compared the percentage of patients in selected KDIGO and KDOQI targets regarding Hb, ferritin, PTH, weekly dialysis time, and eKt/V in men and women.

### 2.2. Definitions

Cardiovascular disease (CVD) was defined as the diagnosis of at least one of the following: heart failure, coronary artery disease, peripheral artery disease, cerebrovascular disease. Hypertension was defined as pre-dialysis BP ≥ 140/90 mm Hg and/or using antihypertensive medication. Apparent treatment resistant hypertension was defined as pre-dialysis BP ≥ 140/90 while on 3 or more antihypertensive drugs or using ≥4 antihypertensive drugs irrespective of BP values. Obesity was defined as BMI more than 30 kg/m^2^ according to the WHO (https://www.who.int/health-topics/obesity#tab=tab_1, accessed 15 October 2026).

### 2.3. Statistical Analysis

Continuous variables were assessed for distributional characteristics. Variables with approximately normal distributions are presented as mean ± standard deviation (SD), whereas variables with markedly skewed distributions (skewness > 1 or <−1) are presented as median and interquartile range (IQR). Categorical variables are presented as counts and percentages. Unadjusted comparisons between men and women were performed using Student’s *t*-test for normally distributed continuous variables, the Mann–Whitney U test for skewed continuous variables, and the chi-square test for categorical variables. To account for potential confounding by age and comorbidities, adjusted analyses were performed using multivariable regression models. Variables were selected based on a combination of clinical relevance and observed baseline imbalance. Age, cardiovascular disease, and diabetes were chosen because they are recognized determinants of patient outcomes and were considered the most important potential confounders in our cohort. For continuous outcomes, linear regression models were fitted, whereas for categorical outcomes, logistic regression models were used. Age, cardiovascular disease, and diabetes mellitus were included as covariates in all adjusted analyses.

Propensity score matching (PSM) was performed as an additional method to reduce baseline differences between men and women. Propensity scores were estimated using a logistic regression model with sex as the dependent variable and age, cardiovascular disease, and diabetes mellitus as predictor variables. Matching was performed using nearest neighbor 1:1 matching without replacement, as implemented in the MatchIt package in R [27]. Covariate balance was assessed using standardized mean differences (SMDs). Before matching, SMDs were 0.199 for age, 0.056 for cardiovascular disease, and 0.066 for diabetes. After matching, SMDs decreased to 0.023, 0.031, and 0.052, respectively, indicating adequate covariate balance (all SMDs < 0.10). Statistical comparisons in the matched cohort were performed using Student’s *t*-test or the Mann–Whitney U test for continuous variables, as appropriate, and the chi-square test for categorical variables. Missing values were not imputed, and no substitution methods (including mean value imputation) were applied. Statistical analyses were performed using available case data, with the number of observations reported where appropriate. All statistical analyses were conducted using R version 4.1.2 (R Foundation for Statistical Computing, Vienna, Austria). Propensity score matching was performed using the MatchIt package. All statistical tests were two-sided, and a *p*-value <0.05 was considered statistically significant.

### 2.4. Ethics

Ethical approval was waived by the local Ethics Committee since the database was collected for the use of a health care provider and all data were fully anonymized before the authors had any access to them. Moreover, there was no direct patient contact whatsoever. The institutional review board at the Medical University of Warsaw, Poland, granted a positive opinion for this study AKBE/16/2023.

## 3. Results

The characteristics of all patients, men, and women are given in the Table 1.

Unadjusted and adjusted analysis revealed that men when compared with women were younger and had significantly lower BMI, higher dry body weight and bioimpedance. The analysis disclosed higher prevalence of CV disease, CAD, PAD, hypertension, higher BP values, higher number of antihypertensive drugs used, higher prevalence of aTRH, and lower incidence of obesity and diabetes in men compared to women. Regarding parameters related to the dialysis, men compared to women more often had arteriovenous fistula, lower dialysis vintage, higher time of dialysis, lower eKT/V and higher UF. In adjusted analysis, prevalence of hypertension and aTRH as well as systolic and diastolic blood pressure values were significantly higher in men than women. In addition, regarding parameters related to the dialysis, men more often had arteriovenous fistula as vascular access, shorter dialysis vintage, higher time of dialysis, lower eKT/V and higher UF and IDWG (Table 2). After indexing dry body weight, UFi was higher and but IDWG was lower in men. Charlson comorbidity score, CHF, cerebrovascular disease, active status on Tx list, and HR were not significantly different in unadjusted and adjusted analysis.

More men than women fulfilled KDIGO targets regarding Hb > 11.5 g/dL, PTH in the range 130–585 pg/mL (more than 2–9 upper normal limit for the laboratory i.e., 2 × 65 ng/dL–9 × 65 ng/dL), (Table 3). On the contrary, more women fulfilled targets for eKt/V > 1.2 (80.5% vs. 64.9%). Similar findings were found in propensity score analysis (Table 4).

## 4. Discussion

Our study showed the differences between men and women in a large, unselected hemodialyzed population throughout the country. Most of the observed significant differences between men and women were small, and probably without clinical significance. However, it is worth underlining that men were on average a few years younger, with higher prevalence of CV disease, including CAD and PAD, and lower prevalence of diabetes than women. Men also had higher dry body mass and OH/ECW. The median dialysis vintage for male patients was found to be 38 months, in contrast to 42 months for female patients (*p* < 0.001).

Sleiman et al. [28] conducted a narrative literature review to determine whether gender inequalities were found in CKD patients in general and in access to different treatment modalities in particular. They established that a greater proportion of male patients progress to end-stage renal disease and require dialysis treatment [26]. In the present study, males constituted 60% of the unselected population, a finding that is consistent with the findings by Sleiman et al. [28]. The most extensive study to date that makes a comparison between male and female patients on hemodialysis is an analysis of the Dialysis Outcomes and Practice Patterns Study (DOPPS) [9], which includes data from 200,637 patients from 12 countries participating in the study. However, the full dataset pertained to 35,964 patients selected from each center [9]. For comparisons with DOPPS results, they used data from national hemodialysis registries in Australia/New Zealand, Canada, Japan, Belgium, France, Spain, Sweden, the United Kingdom and the United States. They also provided patient characteristics by sex and region; however, Europe was combined with Australia/New Zealand and compared with North America and Japan. In a manner consistent with the demographic profile of the population, male subjects in the DOPPS cohort from Europe and Australia/New Zealand (*n* = 7836 men and *n* = 5647 women) exhibited a mean age that was lower than that of their female counterparts (63.4 vs. 64 years). Additionally, they were less prone to obesity (12.1% vs. 18.5%), had a higher prevalence of coronary artery disease (46.6% vs. 37.7%), cerebrovascular disease (18.2% vs. 16.4%), hypertension (80% vs. 77%), chronic heart failure (30.0% vs. 27.8%), and peripheral artery disease (34.2% vs. 24.8%), and a lower prevalence of diabetes (28.6% vs. 30.2%). The higher proportion of hemodialysis patients who were male relative to female (62% men in Europe and Australia/New Zealand) was also reported in the DOPPS study [9]. The underlying rationale for this phenomenon is hypothesized to be the accelerated progression of CKD to end-stage kidney disease [29], compounded by the heightened prevalence of hypertension [9]. To date, the DOPPS has provided the most extensive data on hemodialysis in relation to sex. However, it should be noted that these data were prospectively collected between June 1996 and March 2012. Neither Poland nor any Central and Eastern European country participated in DOPPS study. This is despite the fact that after more than 10 years, similar disparities in prevalence of most common comorbidities were found in our study, with higher prevalence of hypertension reaching 91.8% in men and 89% in women. In a European multicenter study, age, body mass index, Charlson comorbidity index (7.0 in men vs. 6.9 in women), and the proportion of patients with diabetes mellitus (25.5% vs. 29.4%) were similar (in women and men [11]), whereas in our study, Charlson comorbidity index was 4.20 in men and 4.12 in women, reflecting a healthier population. In a recent paper published in 2026, the data derived for 2022 from the population-based European Renal Association (ERA) Registry and the United States Renal Data System (USRDS) were compared. The results showed that the United States had a notably higher incidence, prevalence, kidney transplantation rate and mortality of kidney renal transplantation (KRT) compared with Europe [12]. Nevertheless, the disparities between Europe and the US manifested to a greater extent among women than among men [12]. In detail, fewer women initiating KRT than men (100 vs. 194 per million population (pmp) in Europe, and 320 vs. 459 pmp in USA), incidence of KRT for women was lower in Europe (35%) than in the USA (41%), fewer women are dialyzed relative to men (901 vs. 1549 pmp in Europe and 2006 vs. 2890 pmp in USA) and prevalence of women on KRT was lower in Europe (38%) than in the USA (41%). Transplantation rate in women is lower than in men (33 vs. 59 pmp in Europe and 62 vs. 97 pmp in USA). The same applies for mortality. However, no data on comorbidities, vascular access, or dialysis-related parameters were provided. Furthermore, elderly women were more likely to opt for palliative care as opposed to invasive HD [30]. In male patients, the prevalence of some comorbidities such as hypertension, and cardiovascular disease was found to exceed that observed in female patients. The rationale for this phenomenon pertains to the less healthy lifestyle choices of the male demographic, characterized by a higher prevalence of smoking, elevated alcohol consumption, and diminished physical activity. The study found that males exhibited persistently elevated blood pressure levels and a higher incidence of apparent treatment-resistant hypertension, despite being administered a greater quantity of antihypertensive medications. These findings may suggest a higher cardiovascular risk profile among male HD patients and warrant further investigation into sex-specific determinants of volume control and blood pressure regulation.

The markedly diminished prevalence of AVF utilization among women remained consistent following matching procedures, thereby corroborating earlier international reports. A study undertaken in 2003 in the US by Caplin et al. [31] found that the prevalence of arteriovenous fistulas was similar in men and women. Furthermore, the percentage of fistulas used at the initiation of hemodialysis therapy was also found to be similar in both sexes. The authors of the study suggested that the observed differences were attributable to physician practice patterns, rather than anatomic differences between men and women [31]. Nevertheless, a more recent European multicenter study involving 1247 patients revealed that native AVF was present in 73% of female patients and 80% of male patients [11]. This is the only multicenter study with Polish dialysis units. DaVita population (Polish and Portugal data were analyzed and presented together) was older than our FMC population (men, mean age 67.7 years, women 68.3 years vs. 64.1 vs. 66.9 years, respectively).

They found sex-specific differences in terms of body weight and the prescribed dialysis blood flow rate, single-pool eKt/V, and hemoglobin was arteriovenous fistula as vascular access. However, a number of women or percentage is not provided. In addition, DaVita Poland provided data on 517 patients from seven dialysis centers, whereas FMC provided data on 5778 from 73 centers, making our population more representative. The percentage of AVF is much higher in DaVita facilities; it may be also because the number of centers was limited and data were gathered before COVID-19, while our dataset was from 2022. These findings suggest that barriers to AVF creation and maintenance in women remain present despite increasing awareness of sex disparities in vascular access care.

As demonstrated by Markell et al. [32] in the United States Renal Database System, women are less likely to undergo the creation of an arteriovenous fistula, the preferred vascular access for dialysis, partly due to the misconception that surgery is more challenging for women due to their smaller vascular diameters. As demonstrated by Almasri et al. [33] in a systematic review and meta-analysis, discrepancies in vessel size and quality, along with elevated obstruction rates and a heightened occurrence of infectious complications, were observed in female subjects.

Regarding HD parameters, it has been observed that dialysis vintage is longer for female patients, while eKt/V is higher in the female population relative to the male population. As demonstrated in the DOPPS data, a greater proportion of female subjects attained eKt/V levels more than 1.2, a finding that aligns with the results obtained in this study. It has been hypothesized that higher eKt/V levels in female patients may be indicative of enhanced treatment adequacy and quality in patients receiving dialysis. This phenomenon may be attributable to the higher percentage of fat tissue observed in women compared to men. Consequently, the calculated volume (V) and the V/BSA ratio were found to be lower [34]. It is also conceivable that this calculations may provide a rationale for an overestimation of Kt/V in women [34]. Conversely, however, a heightened high metabolic rate compartment in women may result in an escalated synthesis of uremic toxins. Consequently, the calculation of the dialysis dose based on the V value may result in an underestimation of the dialysis time required to eliminate uremic toxins, including medium-sized molecules, phosphorus, and protein-bound substances, in women [35,36]. A higher UF volume and IDWG have been observed in male patients, which is indicative of higher dry body mass. However, DOPPS data has reported an IDWG (percent body weight) of 3.5 for both sexes [9]. In a European multicenter study, dialysis vintage, treatment time, mean interdialytic body weight gain (IDBWG given as percentage per body weight) were similar, in both women and men (2.2 vs. 2.4), while single-pool eKt/V was significantly higher in women (1.9 vs. 1.8) [11].

In the present study, the mean hemoglobin concentration was found to be significantly lower in the female subjects than in the male subjects. As reported, dialysis overdose or administration of larger-than-needed amounts of erythropoietin-stimulating agents among women are attributed to extrapolating men’s therapeutic dosing to women [37,38]. Female patients demonstrate a lower probability of meeting targets for hemoglobin (Hb) levels > 11.5 g/dL. In a European multicenter study, the proportion of patients with Hb between 10–12 g/dL and ≥12 g/dL was similar in women and men in all age groups [11]. If we used the same cut-offs (Hb between 10–12 g/dL and ≥12 g/d, PTH between 150–600 pg/mL, ferritin > 200 ug/L, ferritin > 800 ug/L) as in this study, even after adjustment, significant differences between sexes persisted in our population. The European multicenter study only refers to a position statement from Kidney Disease: Improving Global Outcomes from 2008 [39].

A salient finding of the present study is the absence of any statistically significant differences in active waitlisting for kidney transplantation according to sex. This observation stands in contrast to numerous reports from North America that document a lower access to transplantation among women, particularly in older age groups. In previous research conducted in the US, Bloembergen et al. [40], using an existing data set from the Michigan Kidney Registry, supplemented with data received from the Organ Procurement Agency of Michigan, reported that in all, 5026 incident ESRD patients (less than 65 years) 44.3% were female. Among 1626 actively waitlisted patients, 40.1% were female; 823 of these received a transplant (37.7% female). After adjustment for age, race, and diagnosis, females were 25% less likely to receive a cadaveric transplant than males. Schaubel et al. [41], using data from the Canadian Organ Replacement Register, compared transplantation rates between 20,131 adult men and 13,458 women aged 20 years or older in a setting in which equal access to medical treatment is assumed. They reported that men experienced 20% greater covariable-adjusted kidney transplantation rates relative to women.

A study of data from the USRDS and United Network for Organ Sharing (UNOS) revealed that, of the 563,197 patients initiated on dialysis between 2000 and 2005, a mere 14% were listed on the waiting list [42]. A lack of discrepancy was observed in terms of access to transplantation for women within the 18–45 age range. However, a notable disparity emerged in access to transplantation for women aged 65–75. This discrepancy remained consistent following adjustment for comorbidities [42].

In the most recent studies conducted in the US [43] and Canada [44], these findings were corroborated. Smothers et al. [43] studied 45,015 adults (18–80 years) initiating dialysis in three US states of Georgia, North Carolina, or South Carolina (2012–2016), and data from the United States Renal Data System were linked to the Early Transplant Access Registry, with follow-up to December 2017. They found that in the southeast, in fully adjusted models (age, race/ethnicity, and obesity), women (vs. men) were 14% less likely to be referred. Kim et al. [44] performed an observational study, where they prospectively identified referrals for kidney transplant in adult patients between June 2010 and May 2013 in 12 Canadian transplant centers, and linked these data to information on incident dialysis patients in a national registry Canadian Organ Replacement Register. Among dialysis patients, men made up the majority (from 57.5% in Manitoba to 63,4% in British Columbia). Older age, female sex, diabetes-related ESKD (compared with glomerular disease—or hypertension-related ESKD), the presence of comorbid disease conditions, pre-dialysis nephrology care > 12.0 months, and receipt of dialysis in a dialysis center > 100 km from a transplant center were associated with a lower relative hazard for kidney transplant referral. However, in both papers [42,43], differences in other parameters were not studied in relation to sex.

Ahearn et al. [45] performed a retrospective cohort study of 1,478,037 adults who developed kidney failure between 2005 and 2017 according to the United States Renal Data System; among them, 271,111 were registered on the waitlist and 89,574 underwent deceased donor transplantation. They examined the association between sex and either access to waitlist registration or deceased donor kidney transplantation and tested for interaction between sex and the attributed cause of kidney failure using adjusted models. They found that women with end-stage kidney failure due to type 2 diabetes had 27% lower access to the kidney transplant waitlist and 11% lower access to deceased donor transplantation after waitlisting compared with men. In a recent study, Al Ammary et al. [46] performed a secondary analysis of a prospectively maintained cohort of 54,889 adult first-time kidney transplant candidates in California (1 January 2010 to 31 December 2024) using the Scientific Registry of Transplant Recipients (SRTR). They found that in the dialysis population (*n* = 38,779), in every race and ethnicity (self-reported), Black, Hispanic, Asian, and White/Other, females were the minority (black—37%; Asian 37%; Hispanic—34%; white—34%). In demographic and health characteristics, age, BMI, and prevalence of comorbidities were provided in relation to race and ethnicity but not to sex.

The findings of the present study demonstrate that there was no gender disparity in terms of the probability of being placed on an active waitlist, with both female and male subjects exhibiting equivalent prospects. Data on transplantation access in a European multicenter study were not provided [11].

In the most recent analysis from the European Committee on Organ Transplantation of the Council of Europe (CD-P-TO), published in 2026, a total number of 122,197 patients from 26 CD-P-TO Member States (Albania, Austria, Belgium, Croatia, Cyprus, Czech Republic, Denmark, Estonia, France, Germany, Hungary, Ireland, Italy, Latvia, Lithuania, Luxembourg, Moldova, the Netherlands, Portugal, Slovak Republic, Slovenia, Spain, Sweden, Switzerland, Turkey and the United Kingdom) and three observer countries (Armenia, Georgia and Israel) were recorded as being on the waiting list in Member States that participated in the survey [47]. Amongst the 91,070 potential kidney transplant recipients, 61% were male. Poland was included in the analysis. In our study, despite a non-significant difference in being actively waitlisted, among 449 potential kidney transplant recipients, 63% were male, similarly to CD-P-TO data.

In a recent study, Gompers et al. [48] evaluated the awareness of transplant staff regarding sex disparities and the female evaluation for kidney transplantation in 48 transplant centers located in 13 US states. The authors emphasized that sex disparities in kidney transplant access may be attributable to biased perceptions of women’s frailty, insurance access, lack of social support, and candidate self-selection. In addition, in the most recent study by Hermida et al. [49], male sex was modestly associated with early rejection in highly sensitized recipients. Moreover, they also reported that the impact of epitope-level HLA (human leukocyte antigen) mismatch on antibody-mediated rejection risk may differ between sexes, particularly at the HLA-DQB1 locus. It may be of paramount importance, as antibody-mediated rejection emerged as the main driver of long-term graft loss.

The population under study constituted one-third of all patients receiving dialysis treatment in Poland. It is important to emphasize that, despite the relatively low number of actively waitlisted patients (approximately 7%), women had equal access to kidney transplantation. This contrasts with the findings of previous studies [39,40,50], including DOPPS [9], in which a greater proportion of male patients were transplanted during the observation period.

It is important to note that, despite the substantial statistical significance of numerous sex-related differences that was attributable to the considerable sample size, the absolute magnitude of several of the observed differences was modest. Consequently, the clinical significance of certain findings should be interpreted with caution, whereas variations in vascular access, blood pressure control, and transplantation access appear to be more clinically meaningful.

### Limitations

The dataset is limited to Fresenius Medical Care. However, of the 278 dialysis units in Poland, 98 were public and 180 were non-public, including 73 owned by FMC, where one-third of all hemodialysis patients were treated. Moreover, given the presence of dialysis units in both small and large cities, and the uniform distribution of FMC units across all voivodeships, it can be concluded that there are no observable geographical or center-specific differences.

Furthermore, the dataset under consideration constitutes a substantial number of patients who have undergone dialysis in accordance with the standard protocol, employing the same hardware apparatus, and as part of the operations of one of the most substantial European providers. During the analysis, several factors potentially of importance were not included. These included marital status, employment status and level of education, as well as smoking. The influence of sex on survival was not the object of our assessment. Further limitations of the study included the retrospective and observational nature of the analyses and the absence of mortality data. The database had been primarily designed for reimbursement purposes, and as such, it did not include mortality data. The selection of age, cardiovascular disease, and diabetes was made a priori for the purpose of adjusted analyses and propensity score matching. This selection was made based on their well-established associations with prognosis and clinical status in this patient population. Furthermore, these variables exhibited a meaningful baseline imbalance between the study groups in the unadjusted analysis, providing additional justification for their inclusion in the propensity score model. To address concerns regarding data completeness, it was necessary to add information on the number of available observations and the proportion of missing data for each variable. Missing values were not imputed, and no substitution methods (including mean value imputation) were applied. The issue of sexual disparities within the HD population is not a recent phenomenon, as national data have been accessible for multiple decades in various countries. However, it has not been explicitly addressed in such a direct manner as in the present analysis, which utilized a substantial and therefore representative sample size in Poland. The present study utilized real-world data from FMC, which was published for the first time regarding the prevalence of hypertension [51] and the vaccination rate for the novel coronavirus [52]. It is recognized that observed sex differences should be interpreted with caution. In addition, data on sex-related differences in Central and Eastern Europe, including Poland, are very scarce; therefore, our large national sample size, standardized care in dialysis units, and standardized reporting system are strengths in the evaluation of real-world data in our country. Conversely, these results offer a comprehensive insight into the current landscape of dialysis therapy in Poland.

## 5. Conclusions

In this extensive nationwide cohort of HD patients, there were notable differences between male and female patients with respect to several demographic, dialysis-related, and biochemical characteristics. Following adjustment for age and comorbidity burden, the most consistent sex-related differences were observed in relation to vascular access, blood pressure control, dialysis adequacy indices, and anemia–mineral bone disorder parameters. Notably, there was no observed discrepancy in active waitlisting for kidney transplantation. The findings of this study indicate that while biological and treatment-related differences between sexes persist in contemporary hemodialysis practice, access to transplantation appears equitable within this population. This extensive cohort, along with the comprehensive set of dialysis-related parameters, offers a potentially valuable foundation for elucidating sex-specific characteristics. Consequently, the dataset has the potential to serve as a significant reference point for future research. Based on the present data, better blood pressure control should be considered in men, and better fluid control in women. Efforts should be undertaken for timely and effective AV fistula creation as the best vascular access in women. The knowledge base on sex differences in the management of hemodialysis patients has expanded over the past years; however, the existence of systematic underlying mechanisms of sex differences in dialysis practices as well as in CKD should be explored further for better outcomes in these vulnerable populations.

## Figures and Tables

**Table 1:** Characteristics of all patients; comparison of men and women.

	All pts.	Missing Data *n* (%) of Total Cohort	Men	Women	*p*	Adjusted * *p*
	***N* = 5879**		***N* = 3519**	***N* = 2360**		
Age [yrs.]	65.2 (14.2)	0	64.1 (14.2)	66.9 (14.1)	<0.001	<0.001
BMI [kg/m^2^]	27.5 (6.02)	0	27.4 (5.48)	27.8 (6.72)	0.012	0.007
Dry body mass [kg]	75.9 (18.1)	0	80.2 (17.4)	69.4 (17.3)	<0.001	<0.001
Obesity	1791 (30.5%)	0	1010 (28.7%)	781 (33.1%)	<0.001	<0.001
OH/ECW	11.4 (11.1)	753 (13)	12.4 (10.6)	10.0 (11.7)	<0.001	<0.001
Charlson comorbidity score	4.17 (1.90)	0	4.20 (1.93)	4.12 (1.86)	0.101	0.019
CVD	3564 (60.6%)	0	2172 (61.7%)	1392 (59.0%)	0.038	1.000
CHF	2114 (36.0%)	0	1285 (36.5%)	829 (35.1%)	0.289	0.846
CAD	1678 (28.5%)	0	1045 (29.7%)	633 (26.8%)	0.018	0.012
PAD	1708 (29.1%)	0	1071 (30.4%)	637 (27.0%)	0.005	0.002
Cerebrovascular	754 (12.8%)	0	443 (12.6%)	311 (13.2%)	0.534	0.294
Diabetes mellitus	2068 (35.2%)	0	1193 (33.9%)	875 (37.1%)	0.013	1.000
Active on transplant list	449 (7.64%)	0	282 (8.01%)	167 (7.08%)	0.202	0.850
SBP [mmHg]	140.8 (17.0)	0	141.6 (17.2)	139.6 (16.7)	<0.001	<0.001
DBP [mmHg]	76.2 (9.71)	0	77.1 (9.89)	74.8 (9.29)	<0.001	<0.001
HR [1/min]	74.0 (8.51)	0	73.9 (8.67)	74.1 (8.25)	0.601	0.601
No. of antihypertensives	2.33 (1.51)	0	2.37 (1.52)	2.26 (1.49)	0.003	0.002
Hypertension	5330 (90.7%)	0	3230 (91.8%)	2100 (89.0%)	<0.001	0.002
aTRH	2179 (37.1%)	0	1369 (38.9%)	810 (34.3%)	<0.001	0.008
Hb [g/dL]	10.8 (1.36)	193 (3)	10.9 (1.41)	10.7 (1.27)	<0.001	0.001
Ferritin [µg/L]	516 [242;935]	4038 (69)	450 [210;871]	610 [292;1052]	<0.001	0.003
PTH [ng/mL]	276 [149;477]	281 (5)	279 [153;475]	270 [140;482]	0.325	<0.001
Access AVF	3325 (56.6%)	0	2137 (60.7%)	1188 (50.3%)	<0.001	<0.001
Dialysis vintage [months]	40.0 [16.0;79.0]	223 (4)	38.0 [15.0;75.0]	42.0 [16.0;86.0]	0.001	<0.001
Dialysis time [minutes per week]	729 [720;744]	175 (3)	732 [721;750]	727 [718;737]	<0.001	0.019
eKt/V	1.37 [1.22;1.52]	383 (7)	1.31 [1.17;1.43]	1.48 [1.32;1.63]	<0.001	<0.001
UF [mL]	2069 (914)	0	2227 (945)	1834 (810)	<0.001	<0.001
UFi	0.29 (0.13)	12	0.29 (0.12)	0.28 (0.13)	0.008	0.006
IDWG [kg]	2.80 [1.70;3.90]	715 (12)	2.80 [1.80;4.00]	2.60 [1.60;3.80]	<0.001	0.012
IDWGi [%]	3.63 [2.11;5.69]	715 (12)	3.55 [2.17;5.39]	3.75 [2.00;6.24]	0.006	<0.001

BMI—body mass index; OH/ECW, where OH—overhydration in L and ECW—extracellular water in L; Charlson—Charlson comorbidity index score; CVD—cardiovascular disease; CHF—congestive heart failure; CAD—coronary artery disease; PAD—peripheral artery disease; cerebrovascular—cerebrovascular disease; SBP—systolic blood pressure; DBP—diastolic blood pressure; HR—heart rate; PTH—parathormone; AVF—arteriovenous fistula; dialysis time—effective weekly dialysis time; eKt/V—equilibrated Kt/V; UF—ultrafiltration; IDWG—interdialytic weight gain; IDWGi—IDWG indexed over dry body mass. * *p* adjusted for age, CV disease, diabetes. Data provided are number and percentage, means (SD) or medians [IQR].

**Table 2:** Characteristics of all patients, comparison of men and women in the propensity score matched * groups.

	All pts.	Men	Women	*p*
	***N* = 4720**	***N* = 2360**	***N* = 2360**	
Age [yrs.]	66.7 (14.0)	66.6 (13.8)	66.9 (14.1)	0.419
BMI [kg/m^2^]	27.6 (6.09)	27.4 (5.37)	27.8 (6.72)	0.043
Dry body mass [kg]	74.7 (18.0)	80.0 (17.0)	69.4 (17.3)	<0.001
Obesity	1463 (31.0%)	682 (28.9%)	781 (33.1%)	0.002
OH/ECW	11.1 (11.4)	12.2 (10.9)	10.0 (11.7)	<0.001
Charlson comorbidity score	4.15 (1.90)	4.18 (1.94)	4.12 (1.86)	0.312
CVD	2759 (58.5%)	1367 (57.9%)	1392 (59.0%)	0.478
CHF	1650 (35.0%)	821 (34.8%)	829 (35.1%)	0.831
CAD	1309 (27.7%)	676 (28.6%)	633 (26.8%)	0.172
PAD	1333 (28.2%)	696 (29.5%)	637 (27.0%)	0.061
Cerebrovascular	584 (12.4%)	273 (11.6%)	311 (13.2%)	0.102
Diabetes mellitus	1706 (36.1%)	831 (35.2%)	875 (37.1%)	0.193
Active on transplant list	338 (7.16%)	171 (7.25%)	167 (7.08%)	0.866
SBP [mmHg]	140.1 (17.0)	141.6 (17.2)	140.6 (16.7)	0.012
DBP [mmHg]	75.5 (9.51)	76.2 (9.68)	74.8 (9.29)	<0.001
HR [1/min]	73.9 (8.50)	73.7 (8.74)	74.1 (8.25)	0.183
No. of antihypertensives	2.28 (1.49)	2.31 (1.49)	2.26 (1.49)	0.223
Hypertension	4251 (90.1%)	2151 (91.1%)	2100 (89.0%)	0.015
ATRH	1683 (35.7%)	873 (37.0%)	810 (34.3%)	0.019
Hb [g/dL]	10.8 (1.34)	10.9 (1.40)	10.7 (1.27)	0.001
Ferritin [µg/L]	536 [253;967]	463 [210;902]	610 [292;1052]	<0.001
PTH [pg/mL]	268 [143;464]	265 [145;451]	270 [140;482]	0.395
Access AVF	2591 (54.9%)	1403 (59.4%)	1188 (50.3%)	<0.001
Dialysis vintage [months]	40.0 [16.0;79.0]	37.0 [15.0;73.0]	42.0 [16.0;86.0]	<0.001
Dialysis time [minutes per week]	729 [720;742]	731 [720;749]	727 [718;737]	<0.001
eKt/V	1.39 [1.23;1.55]	1.31 [1.17;1.43]	1.48 [1.32;1.63]	<0.001
UF [mL]	2005 (890)	2176 (933)	1834 (810)	<0.001
UFi	0.28 (0.13)	0.28 (0.12)	0.28 (0.13)	0.435
IDWG [kg]	2.70 [1.60;3.90]	2.80 [1.70;3.90]	2.60 [1.60;3.80]	0.006
IDWGi [%]	3.61 [2.05;5.76]	3.45 [2.10;5.34]	3.75 [2.00;6.24]	<0.001

* matched for age, CV disease, diabetes. BMI—body mass index; OH/ECW, where OH—overhydration in L and ECW—extracellular water in L; Charlson—Charlson comorbidity index score; CVD—cardiovascular disease; CHF—congestive heart failure; CAD—coronary artery disease; PAD—peripheral artery disease; cerebrovascular—cerebrovascular disease; SBP—systolic blood pressure; DBP—diastolic blood pressure; HR—heart rate; PTH—parathormone; AVF—arteriovenous fistula; dialysis time—effective weekly dialysis time; eKt/V—equilibrated Kt/V; UF—ultrafiltration; UFi—UF indexed over dry body mass; IDWG—interdialytic weight gain; IDWGi—IDWG indexed over dry body mass. Data give are number and percentage, means (SD) or medians [IQR].

**Table 3:** Patients in KDIGO /KDOQI targets according to sex.

	ALL	Men	Women	*p*	Adjusted *p*
Hb > 11.5 g/dL	1649 (27.9%)	3519 (29.6%)	600 (25.4%)	<0.001	<0.001
Ferritin < 200 µg/L	383 (6.5%)	267 (7.6%)	116 (4.9%)	<0.001	<0.001
PTH 130–585 pg/mL	3464 (58.9%)	2126 (60.4%)	1338 (56.7%)	0.005	0.004
Dialysis time > 12 h per week	4167 (70.9%)	2565 (72.9%)	1602 (67.9%)	<0.001	<0.001
eKt/V > 1.2	4183 (71.2%)	2283 (64.9%)	1900 (80.5%)	<0.001	<0.001

Hb—hemoglobin; PTH—parathormone; eKt/V—equilibrated Kt/V.

**Table 4:** Patients in KDIGO/KDOQI targets according to sex in matched groups.

	ALL	Men	Women	*p*
Hb > 11.5 g/dL	1297 (27.5%)	697 (29.5%)	600 (25.4%)	0.002
Ferritin < 200 µg/L	295 (6.2%)	179 (7.6%)	116 (4.9%)	<0.001
PTH 130–585 pg/mL	2761 (58.5%)	1423 (60.3%)	1338 (56.7%)	0.013
Dialysis time > 12 h per week	3298 (69.9%)	1696 (71.9%)	1602 (67.9%)	0.003
eKt/V > 1.2	3428 (72.6%)	1528 (64.7%)	1900 (80.5%)	<0.001

Hb—hemoglobin; PTH—parathormone; eKt/V—equilibrated Kt/V.

## Data Availability

The data underlying this article will be shared on reasonable request to the corresponding author.
